# Explicit Logic Circuits Predict Local Properties of the Neocortex's Physiology and Anatomy

**DOI:** 10.1371/journal.pone.0009227

**Published:** 2010-02-16

**Authors:** Lane Yoder

**Affiliations:** Department of Mathematics, University of Hawaii, Kapiolani, Honolulu, Hawaii, United States of America; Newcastle University, United Kingdom

## Abstract

**Background:**

Two previous articles proposed an explicit model of how the brain processes information by its organization of synaptic connections. The family of logic circuits was shown to generate neural correlates of complex psychophysical phenomena in different sensory systems.

**Methodology/Principal Findings:**

Here it is shown that the most cost-effective architectures for these networks produce correlates of electrophysiological brain phenomena and predict major aspects of the anatomical structure and physiological organization of the neocortex. The logic circuits are markedly efficient in several respects and provide the foundation for all of the brain's combinational processing of information.

**Conclusions/Significance:**

At the local level, these networks account for much of the physical structure of the neocortex as well its organization of synaptic connections. Electronic implementations of the logic circuits may be more efficient than current electronic logic arrays in generating both Boolean and fuzzy logic.

## Introduction

Two previous articles [1], [2] presented a family of general logic circuits that exploit neurons' capabilities of excitation and inhibition in varying degrees of intensity. These networks provide fuzzy logic conjunctions, with negations, of any number of propositions [2]. The networks are capable of processing information for a variety of brain functions. To illustrate the networks' capabilities, they were shown to generate neural correlates of several psychophysical phenomena central to color vision and olfaction. These logic circuits will be reviewed briefly.

For each of the logic circuits, there is an architecture that minimizes the total cost of the number of cells, connection length, and cellular packing density while retaining the network's functionality. For a few of the simpler logic circuits, the networks with the fewest cells were presented previously [2] without proof that the number of cells was minimized and without the optimal spatial arrangement of the cells. The cells and connections for the general case, the optimal arrangement of cells, and the optimality arguments given here are new.

The architecture with the optimal number of cells is first described recursively, with a few necessary adjustments. Then it is demonstrated that this architecture minimizes the number of cells while retaining functionality. The spatial arrangement of these cells that minimizes the total connection length is determined by contracting the connections. Finally cellular packing density is increased where it does not appreciably affect connection length.

While the entire optimal form cannot be determined exactly because of certain complexities and unknown factors, enough of its properties are established to reach several conclusions: It is markedly efficient in the three defining cost functions as well as several others. It makes detailed predictions of several major anatomical and physiological aspects of cortical organization. It provides the foundation for all of the brain's combinational processing of information, i.e., logic functions whose outputs depend only on the inputs. A future article will show how neurons can be connected to form dynamic memory elements that provide the building blocks for all of the brain's sequential logic operations, whose outputs are functions of both the current inputs and the past sequence of inputs.

## Analysis

### Recursive AND NOT Conjunctions

The logic circuits that were introduced previously [1], [2] were derived from simple principles. The minimal, known cellular properties of excitation and inhibition are stated explicitly in Table 1. The logic circuits are based on the logic identities given in Table 2. Logic circuits can be constructed in many ways depending on different properties of logic, but two criteria determined the choice of the identities in Table 2: The identities define every logical conjunction in terms of simple X AND NOT Y gates, and they produce logic circuits that generate both classical Boolean logic and fuzzy logic. These criteria were chosen because they exploit two key neural capabilities that follow from the cellular characteristics listed in Table 1: The logical AND NOT gate is compatible with neural excitation and inhibition, and fuzzy logic takes advantage of the large amount of information conveyed in signals that encode varying degrees of stimulation. The networks' fuzzy logic follows directly from the properties of Table 1 and the identities of Table 2 [2]. It is this fuzzy logic that was shown to correspond to neural information processing [1], [2]. There is no obvious reason to expect the logic identities of Table 2 to lead to efficient logic circuits, but it will be shown that optimal architectures based on these identities are quite efficient in several ways.

**Table 1:** Cellular response properties.

1. 1∼0 = 1.	Maximum excitation elicits maximum response.
2. X∼Y = 0 if X 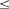 Y.	Inhibition cancels equal or smaller excitation.
3. X∼Y is increasing in X.	Greater excitatory input increases output.
4. X∼Y is decreasing in Y.	Greater inhibitory input decreases output.

The properties of the neural logic circuits follow from the networks' architectures and the minimal, well-known cellular characteristics listed here. If X and Y are two cells' response intensities, X∼Y represents the response intensity of a neuron with excitatory input X and inhibitory input Y. Responses are normalized to be in the interval from 0 to 1.

**Table 2:** Recursive AND NOT Conjunction definitions and responses.

Recursive logic identity for constructing a RANC	Interval measured by the RANC response	Approximate value of the RANC response
1. 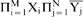 = 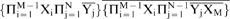	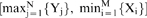	|| 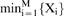 − 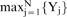 ||
2. 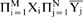 = 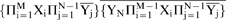		
3. 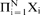 = 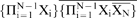	[0, 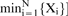	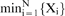
4. 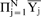 = 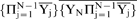	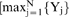 , 1]	1− 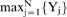

The logic identities in the first column equate every conjunction to a conjunction 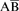. The recursive and reductive identities show how logic circuits can be implemented with neural AND NOT gates using the neural AND NOT property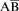 = A∼B. The second column shows the interval measured by the corresponding RANC response. The third column shows the approximate value of the response is the length of the interval. The notation ||b−a|| stands for the length of the interval [a, b] if a<b, and 0 otherwise.

Neuron response intensities are represented by variables X and Y. For cells that transmit all-or-nothing action potentials, the response intensity is the frequency of action potentials. Response intensities are normalized by dividing them by the maximum possible intensity for the given level of adaptation. This puts response intensities in the interval from 0 to 1, with 0 meaning no signal and 1 meaning the maximum intensity. This number is called the *response intensity* or simply the *response* of the receptor or neuron. If the responses of two sensory receptors or neurons are X and Y, the notation *X∼Y* represents the response of a neuron with excitatory input X and inhibitory input Y. For binary inputs of 0 or 1, the properties of Table 1 imply that such a neuron performs the logical AND NOT function. That is, X∼Y = 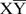 (X AND NOT Y). This response property is called the *neural AND NOT property*.

Since the logic identities of Table 2 are recursive and define a general conjunction in terms of AND NOT gates, the resulting logic circuit is called a *Recursive AND NOT Conjunction* (RANC). For n propositions that have one of two possible truth values, true or false, there are 2^n^ possible combinations of truth values. Each combination corresponds to a conjunction of n propositions. For two propositions X and Y, for example, there are four conjunctions: 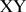, 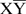, 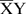, and 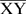. An *n-RANC* produces one or more conjunctions of n propositions. A *single* n-RANC produces one of the conjunctions, and a *Complete* n-RANC produces all of the 2^n^ possible conjunctions. There is no theoretical bound on n.

**Figure 1:**
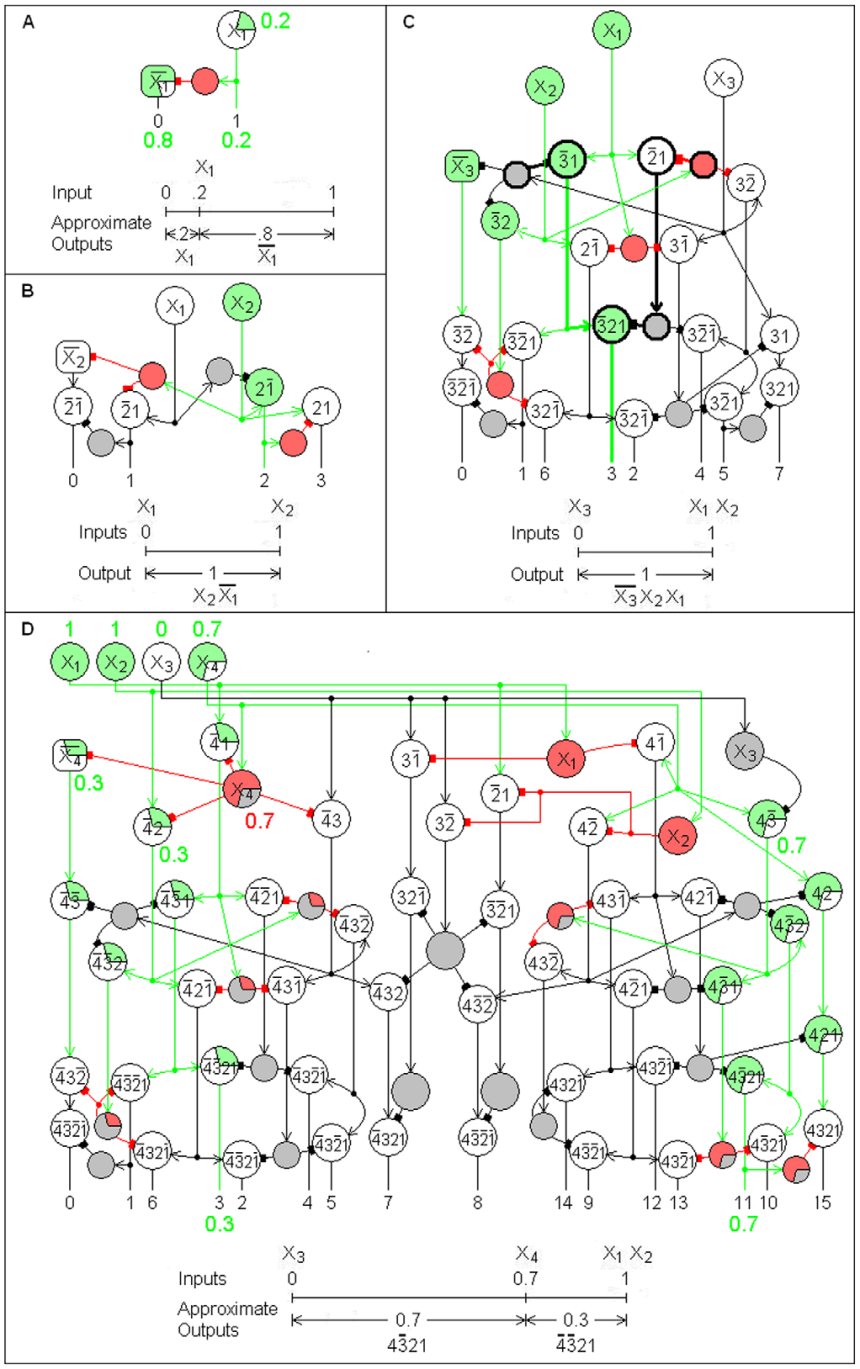
Recursive AND NOT Conjunctions. A Recursive AND NOT Conjunction (RANC) is a general logic circuit that produces conjunctions, with negations, and is defined recursively in terms of AND NOT gates. An n-RANC produces conjunctions of n propositions. A Complete n-RANC produces all conjunctions corresponding to the 2^n^ possible combinations of truth values of n propositions. Examples of Complete n-RANCs are shown here for n = 1–4. The label on each neuron represents its response. The maximum and minimum possible responses 1 and 0 can stand for the logical values true and false, making the network outputs logical functions of the inputs. Arrows indicate excitatory input; blocks indicate inhibitory input. Spontaneously active neurons are square. To illustrate example inputs and outputs, active neurons are colored. Inactive inhibitory cells are shaded. The line graphs below each circuit diagram illustrate the RANC interval measure property and the Boolean and fuzzy logic of the example inputs and outputs.

**Figure 2:**
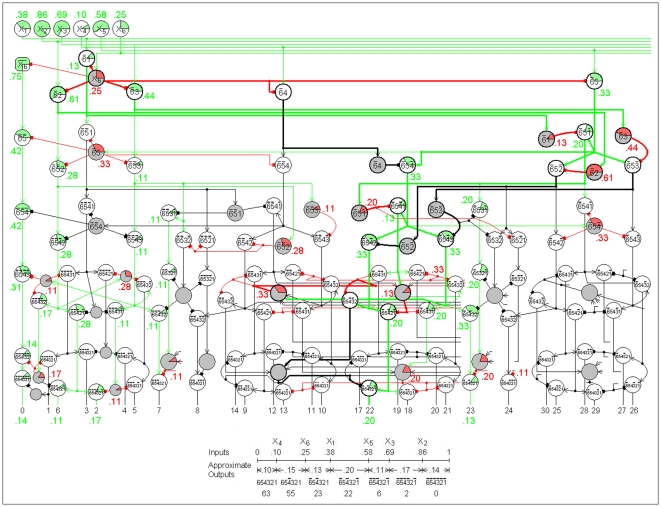
A Recursive AND NOT Conjunction with several inputs. The circuit diagram shows nearly half of a Complete 6-RANC. It illustrates several general properties of BC n-RANCs, including properties that hold for n>4. The second and third columns have shared inhibitory cells. The example inputs produce active regions surrounded by neurons that are inhibited below the resting level. The example inputs also show that information is processed in small columns and is transformed in each layer while passing through the columns in series and in parallel. The diagram illustrates the modular architecture that is determined by the recursive definition of a BC n-RANC. The line graph shows the RANC interval measure property and the fuzzy logic of the approximate outputs. For example, the output number 22, 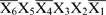 = 0.20, represents the truth value of the conjunction “X_2_, X_3_, and X_5_ are true, and X_1_, X_4_, and X_6_ are false.”

Examples of Complete n-RANCs are shown in Fig. 1 for n = 1, 2, 3, and 4. Fig. 2 shows part of a Complete 6-RANC. To show the cells and their connections clearly, the diagrams are three dimensional and the views are exploded. For now there is no claim that the diagrams are in any way optimal. All of the RANCs are columnar as they are arranged in Figs. 1 and 2. Most of the columns have six excitatory cells in each of the lower two layers, with each layer connected in a closed curve. These are called *hexagonal* columns. Active cells are colored to illustrate example responses.

For brevity, only the conjunctions' subscripts are shown in Figs. 1 and 2. For example, the response 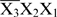 is abbreviated as 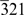. The subscripts are written in descending order to match the standard digit ordering of the numeric labels for the networks' outputs. For example, the binary number 011 equals the decimal number 3. The response 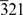 in Fig. 1C is labeled “3” below the network because it has the value 1 if and only if the inputs X_3_, X_2_, X_1_ are 0, 1, 1, respectively. This particular state of RANC cell responses is illustrated by the colors in Fig. 1C. The single 3-RANC that produces this output is illustrated with thick lines. The single 6-RANC for output number 22 in Fig. 2 is also shown in thick lines. The whole number labels for the RANC outputs are meant to provide a short and mnemonic way of referring to the different outputs.

An important RANC property is that the neuron response for the conjunction in the first column of Table 2 is a measure of the corresponding interval in the second column [2]. This means that a RANC response for a conjunction in column 1 of Table 2 increases from 0 to 1 as the endpoints of the corresponding interval in column 2 increase the interval length from 0 to 1. As a measure of the interval, the response is approximately the length of the interval. The accuracy of the approximation depends inversely on the degree of nonlinearity in the neural response function. This *RANC interval measure property* is illustrated in the line graphs in Figs. 1 and 2. The property implies that RANCs perform classical logic functions for inputs that have binary values of 0 and 1 and fuzzy logic for intermediate inputs. From this property it was shown that RANCs generate detailed neural correlates of known psychophysical phenomena [1], [2]. Only one simple consequence of the interval measure property will be used here, and it will be used only once: If a RANC response 
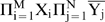
 is positive, then Y_j_<X_i_ for all i, j.

### Best Complete n-RANCs

The number of possible ways of implementing any logic circuit that has more than two or three inputs is quite large. The different architectures vary widely in their efficient use of resources. This is true even under the restrictions of the Table 2 logic identities that define a RANC. For each n there exists at least one Complete n-RANC that minimizes the total cost of the number of cells, total connection length, and cellular packing density while retaining functionality. This network (or any one of the networks if there is more than one) will be called the *Best* Complete n-RANC (BC n-RANC).

Determining the exact architecture and spatial configuration of the BC n-RANC entails potential problems. Although the optimal arrangement of cells in each hexagonal column is fairly straightforward, the arrangement of columns and the arrangement of cells that produce input to the columns are complex. Because of this the complete optimal configuration is not determined here, but enough of its geometric properties are found for the conclusions of this article.

The three cost variables – number of cells, total connection length, and packing density – are not independent. A decrease in one cost can incur an increase in another. In addition, the relative costs of the variables are not known, e.g., the cost of one cell vs. the cost of a one centimeter connection. This means that if a reduction in one cost incurs an increase in another, it cannot be proven whether the tradeoff results in a net gain or loss. It also means there is no way to measure how close any particular network comes to minimizing the total cost.

Some tradeoffs have large asymmetries, where a slight increase in one cost can mean an obviously significant decrease in another cost. Although the cost benefit cannot be measured, it can be argued that the net change is fairly obviously a positive benefit because of the large asymmetry. In a few cases here, the trade affects the network's predictions about the brain. Each of these trades affects only one or two of the network's predictions, and in each case the network that is apparently more cost effective predicts more accurately. There are three such asymmetric trades here. In the next section it is shown that the number of cells is minimized at the cost of connection lengths that are slightly greater than the minimum. Then it is shown that greater functionality can be achieved at the cost of a few more cells than the minimum number. After the cells have been arranged to minimize total connection length for this network, it is shown that an increase in packing density in the upper layers can be gained at the cost of a possible slight increase in connection length.

Fig. 1A–C defines the cells and connections of the BC n-RANC for n = 1, 2, 3. For n>3, the cells and connections of the BC n-RANC are defined recursively in terms of two BC (n−1)-RANCs, with some modifications described below. If the inputs to the BC n-RANC are X_1_, …, X_n_, the inputs to one BC (n−1)-RANC are 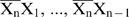, and the inputs to the other are 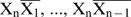. This definition is illustrated in Fig. 1D, which shows a BC 4-RANC that is constructed from two BC 3-RANCs. Fig. 2 shows half of a BC 6-RANC, which means the diagram is a BC 5-RANC with inputs 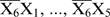. The diagram also shows the BC 5-RANC is formed from two BC 4-RANCs. (To avoid confusion it should be noted that although both RANCs and BC n-RANCs are defined recursively, a RANC is defined by the logic identities of Table 2 but a BC n-RANC is defined more narrowly.)

The recursive definition of a BC n-RANC produces a Complete n-RANC because conjoining a proposition (A) with each of the inputs (X and Y) to an AND NOT gate simply results in conjoining that proposition with the output 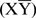: By the neural AND NOT property, (AX)∼(AY) = 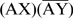, and 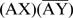 = 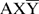 by the algebra of classical logic. The propositions joined to the inputs are passed along through the entire (n−1)-RANCs and are joined to the outputs. The first (n−1)-RANC produces the outputs labeled 0 to 2^n−1^−2, and the second produces outputs 2^n−1^+1 to 2^n^−1.

Some modifications are required in a few parts of this recursive process. Shared cells can reduce resource requirements and are discussed immediately below. Somewhat obvious modifications are also required for the single n-RANCs that produce the outputs labeled 0 and 2^n^−1 so that they are produced according to the logic identities 3 and 4 in Table 2. These n-RANCs are shown on the left and right sides of Fig. 1A–D and the left of Fig. 2.

**Figure 3:**
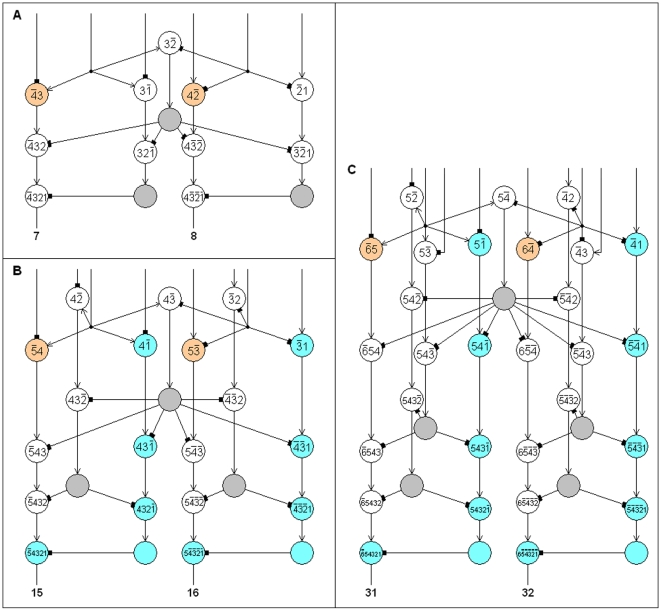
Non-hexagagonal columns. A fourth of a BC n-RANC's outputs are not produced by hexagonal columns. This includes the n-RANCs for the outputs labeled 2^n−1^−1 and 2^n−1^. These n-RANCs are shown in A, B, C for n = 4, 5, 6, respectively. To transform each single n-RANC into an (n+1)-RANC, n excitatory cells and one inhibitory cell are added as shown in blue. Other non-hexagonal n-RANCs are formed from these by the recursive definition of a BC n-RANC. The input cells that are not shown and the cells shown in orange are not part of the networks because they are either inputs to the BC n-RANC or part of other n-RANCs within the BC n-RANC.

The single n-RANCs for the outputs labeled 2^n−1^−1 and 2^n−1^ are constructed differently from the recursive definition of a BC n-RANC because they must conform to equations 1 and 2 in Table 2 to have the interval measure property. These single n-RANCs are in non-hexagonal columns, but they have essentially the same architecture as a single n-RANC that forms part of a hexagonal column. Examples of such columns are shown in Fig. 1D for n = 4 (producing outputs 2^4-1^−1 = 7 and 2^4-1^ = 8) and in Fig. 3 for n = 4, 5, 6. To transform each single n-RANC into an (n+1)-RANC, n excitatory cells and one inhibitory cell are added as shown in blue in Fig. 3. The n-RANCs in Fig. 3B for outputs 15 and 16 are not shown in Fig. 2 so the connections for the second and third hexagonal columns' shared inhibitory cells can be illustrated more clearly.

### Shared Cells

The number of cells required can be reduced by using certain inhibitory cells to serve multiple functions. Such cells can be shared in BC n-RANCs for n>4. As n increases, the number of single n-RANCs served per shared cell increases rapidly. The cost tradeoff in sharing inhibitory cells is a large reduction in the number of cells for slightly longer connections. Because of the large asymmetry in the tradeoff, shared cells are considered part of the BC n-RANCs. This design decision affects two predictions about the brain – the percentage of inhibitory cells and active regions that are surrounded by inhibited regions.

A neuron with no excitatory input normally discharges at a low, baseline level. The effect of inhibitory input on such a cell's response is detectable but so minute that the response can convey little, if any, useful information contained in the input. If two or more sets of neurons have mutually exclusive activity – meaning whenever any cell in one set is active, no cell in any other set is active – then a single inhibitory cell can replace a group consisting of one inhibitory cell from each set. Because the sets are mutually exclusive, only one set uses the cell at any time. The inactive sets have no effect on the inhibitory cell, and the effect of the cell's inhibitory input to the inactive sets is negligible.

One condition that implies mutually exclusive activity in RANCs is 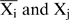 in the response name of every cell in one set and 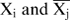 in the response name of every cell in another set. In Fig. 2 for example, the second hexagonal column has 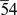 in every cell's response name and the third column has 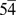. If the set with 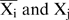 in the response names has an active cell, then X_i_<X_j_ by the interval measure property. Similarly if the set with 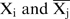 in its response names has an active cell, then X_i_>X_j_. These two states of the inputs are mutually exclusive.

The lower two layers of a RANC hexagonal column require three inhibitory cells for each layer (Figs. 1C, D, 2). This means a set of hexagonal columns with mutually exclusive activity can share six inhibitory cells. For n>3, the recursive definition for BC n-RANCs implies that the response name of every cell in the lower two layers of every hexagonal column has a prefix containing X_4_, …, X_n_, with or without negated components. All the cells in each hexagonal column have the same prefix, and the prefix is different for each column. If two or more columns have the same number of negated components in their prefixes, the columns have mutually exclusive activity because for each such pair of columns there must be at least one pair of inputs X_i_, X_j_ such that 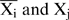 are part of one column's prefix and 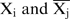 are part of the other column's prefix. Since the prefix containing X_4_, …, X_n_ has length n−3, the number of hexagonal columns with k negated components in its prefix is the number of combinations C(n−3, k), k = 0, 1,…, n−3. There is only one column with no negated component in its prefix (k = 0) and one with all negated components (k = n−3). These two columns cannot share cells.

To sum up this result, for n>4 a BC n-RANC has n−4 sets of mutually exclusive columns. There are C(n−3, k) columns in set number k, k = 1,…, n−4. Each set of columns can share 6 inhibitory cells. For example, a BC 8-RANC has 2^5^ = 32 hexagonal columns, with 4 sets of mutually exclusive columns. These sets consist of 5, 10, 10, and 5 columns and have k = 1, 2, 3, and 4 negated components in their prefixes, respectively. Each of the 4 sets can share 6 inhibitory cells. The total of 24 inhibitory cells is 156 fewer than would be required without sharing of cells. A BC 6-RANC has 2^3^ = 8 hexagonal columns, with 2 sets of 3 mutually exclusive columns. The part of a BC 6-RANC in Fig. 2 shows the shared cells in two of the three hexagonal columns that have two negated components in their prefixes, and the shared cells in one of the three columns that have one negated component in their prefixes. Several other inhibitory cells that are not in hexagonal columns can also be shared, as indicated by the inhibitory cells in Fig. 2 with more than one input and output.

### Modular Architecture

**Figure 4:**
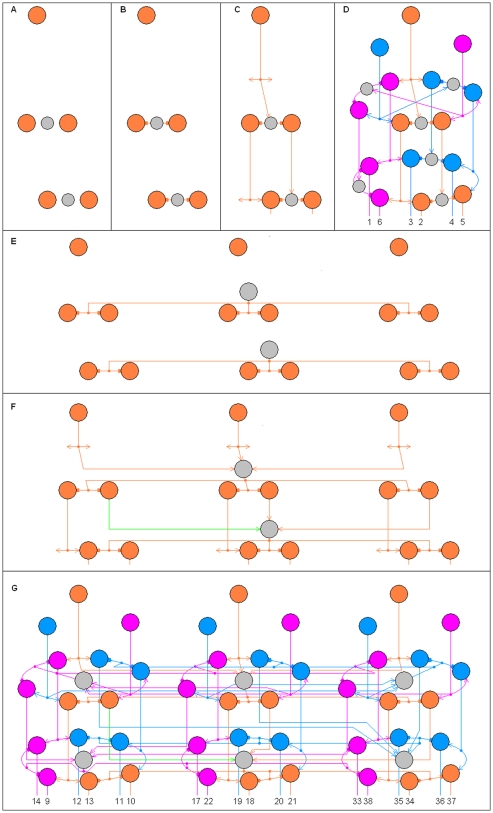
Modular architecture with repeated parts. A RANC hexagonal column begins with unconnected cells in A. Inhibitory connections in B form identical three-cell parts in different layers. Excitatory connections in C combine these parts into a three-layer module. Additional excitatory connections in D join three identical modules to form a column. When several columns share inhibitory cells, as illustrated in Fig. 2, modular construction is nearly as simple. Inhibitory connections in E form identical parts in different layers, and excitatory connections in F combine these parts into a three-layer module. Additional excitatory connections in G join three of these modules to form three columns.

The BC n-RANCs have at least six levels of modular architecture, with each module consisting of a simple combination of a few repeated parts from the previous stage. Fig. 4A–D shows four of these levels: individual cells, identical three-cell parts in different layers, a three-layer module formed from these parts, and a hexagonal column made up of three such modules. If inhibitory cells are shared by several columns, modules can still be constructed with simple combinations of repeated parts. Fig. 4E–G shows this for the construction of three columns. This modular architecture makes assembly of the network considerably simpler than the result appears in Fig. 2. To facilitate clarification of subsequent diagrams, one of the excitatory connections is drawn in green in Fig. 4F, G. Non-hexagonal n-RANCs for the outputs labeled 2^n−1^−1 and 2^n−1^ are also modular, as described previously in the recursive process for constructing them.

At the next n−3 levels, two BC (k-1)-RANCs are combined to form a BC k-RANC, k = 4,…, n, according to the recursive definition of BC n-RANCs. Finally, several BC n-RANCs can be combined to provide the logical structure for functional systems such as olfaction [2]. This hierarchy of modular levels does not necessarily mean the assembly process must proceed sequentially in the order of levels, with the construction of one stage being completed before the next stage begins.

### Number of Cells

Here is it shown that the number of cells in each BC n-RANC is nearly the minimum required for the 2^n^ outputs, and in a sense this number is optimal. Choosing a form that does not absolutely minimize the number of cells affects the prediction of the portion of inhibitory cells in the neocortex. Complete 1- and 2-RANCs are simple enough that optimal architectures are obvious (Fig. 1A, B). For n>2, it will first be shown how a Complete n-RANC can be constructed with slightly fewer cells than what has been defined as the BC n-RANC. Reasons are given for not defining the best one as the one with the fewest cells. With these minor exceptions, it can be demonstrated by exhaustion of all the possibilities that hexagonal columns minimize the number of cells required for a BC n-RANC. The proof is outlined for n = 3, 4. The argument for higher values of n is similar to the proof for n = 4.

Excitatory cells that excite only inhibitory cells could be omitted, and their inputs could be rerouted directly to the inhibitory cells. There are three such cells in the second lowest layer of each hexagonal column and one for each inhibitory cell in each non-hexagonal column. These excitatory cells are included in a BC n-RANC because their responses are m-RANC outputs, m<n, and the information contained in the responses might be usefully projected elsewhere in the brain. For example, the identification of one odorant may depend on a pattern of signals from a certain set of n olfactory receptor types, and identification of another odorant may depend on a pattern of signals from a proper subset of m of those receptor types. In that case, the m-RANC that produces the neural correlate of the second odorant could be a proper subset of the n-RANC that produces the neural correlate of the first.

**Figure 5:**
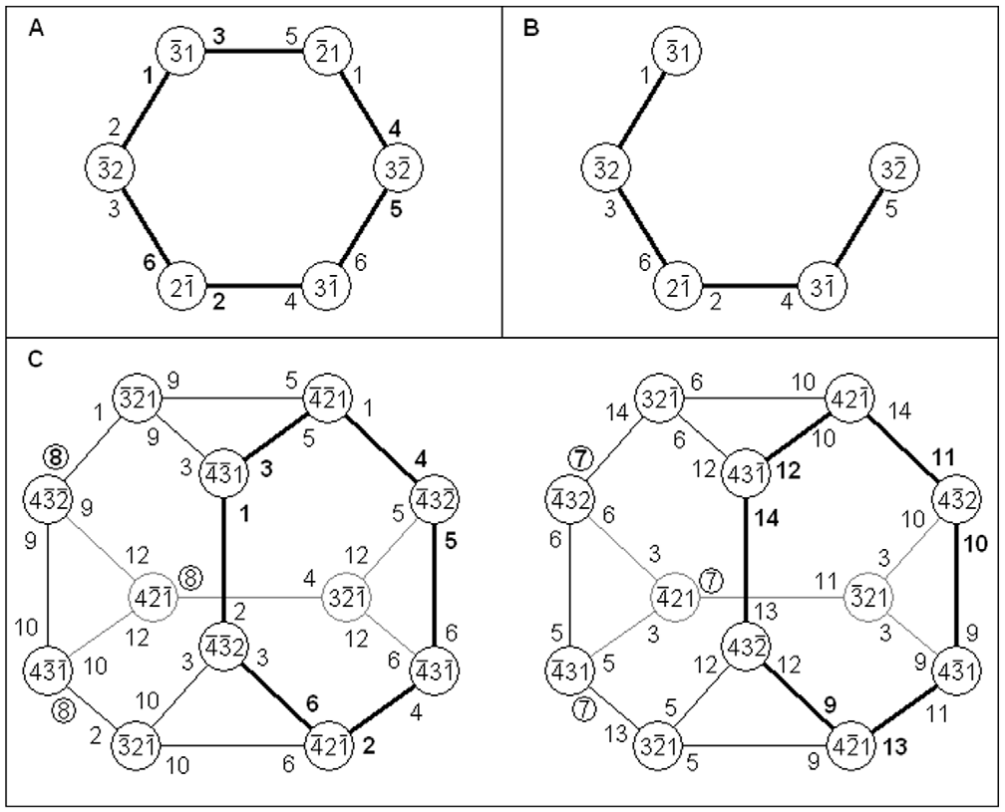
Number of cells required. The directed graphs show that hexagonal columns nearly minimize the number of cells used to construct Complete n-RANCs. Two nodes are linked if they can form a conjunction according to equation 1 or 2 of Table 2. Along each edge, the label representing the response 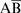 is shown next to node A, and 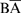 is next to B. The edge labels in bold face show the conjunctions as they are implemented in Fig. 1C, D. The graph in A shows all the ways six single 3-RANCs can be formed, and B shows how six single 3-RANCs can be formed with one less cell. The graph in C shows how 4-RANCs can be formed.

The minimum number of AND NOT gates required for a BC n-RANC is obtained from Fig. 5. The argument does not address the question of how many inhibitory cells are required to produce the AND NOT gates; it was previously shown in the section on shared cells that this number is minimized by sharing inhibitory cells. A few elementary concepts in graph theory are required for the discussion of the Fig. 5 graphs (not to be confused with graphs of functions). Graphs consist of nodes and links, or edges, that connect some pairs of nodes to indicate that the nodes are related in some way. Nodes are usually depicted by circles, and edges by line segments connecting the circles. In a directed graph, the edges have a direction from one node to the other. The edges can be directed in one or both directions.

The directed graphs in Fig. 5 show all possible ways propositions can be paired according to equations 1 and 2 of Table 2 to produce the final outputs of 3- and 4-RANCs. Each node represents a proposition, and two nodes A and B are linked if they can form a conjunction 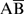 according to either equation 1 or 2 of Table 2. The graphs are directed because each linked pair can form conjunctions in both directions, i.e., as 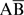 and 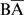. Along each edge, the label representing the conjunction 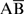 is shown next to node A, and the label representing 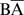 is next to B. For example, at the top of Fig. 5A the two nodes 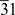 and 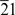 are shown to be linked by a line segment. The edge label 3 next to node 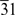 represents the conjunction 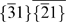 = 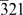. The single 3-RANC that produces this conjunction in this way is shown in thick lines in Fig. 1C.

The hexagonal graph in Fig. 5A shows all possible ways of producing the final outputs of 3-RANCs according to equations 1 and 2 of Table 2. The edge labels show the pairings produce six distinct conjunctions, labeled 1 through 6. As the graph shows, there are many possible ways to connect neurons represented by the nodes to produce the six outputs. All six can be produced efficiently in two different ways using just three cells corresponding to alternate nodes in the graph as excitatory input and cells corresponding to the other three nodes as inhibitory input to the six output cells. Of the two possibilities, the edge labels in boldface show the 3-RANC architectures as they are implemented in Fig. 1C. Both ways require the same number of cells and have identical architectures; the only difference is in which cells provide excitatory input and which provide inhibitory input to the output cells.

Fig. 5B shows that the six conjunctions can be produced with any one of the nodes removed from the hexagon in Fig. 5A. Although this design is a slightly more resource-efficient possibility and applies to all n-RANC hexagonal columns for n>2, it is not incorporated in the BC n-RANC because it only reduces the number of cells by one per hexagonal column, because the cell produces a conjunction that may be useful as described at the beginning of this section, and because removing the cell precludes the modular construction illustrated in Fig. 4. Except for the two possibilities of removing a few cells described here and above, it is clear from a simple check of the finite number of possibilities shown in Fig. 5A that the two equivalent alternatives discussed in the previous paragraph are the most efficient ways of constructing the six single 3-RANCs that can be constructed from equations 1 and 2 of Table 2. Except for the choice between two equivalent alternatives, this architecture uniquely determines all of the cells and connections of the six 3-RANCs.

The graph in Fig. 5C shows all possible ways of producing the final outputs of 4-RANCs according to equations 1 and 2 of Table 2. The graph consists of two disconnected pieces because equations 1 and 2 imply that linked nodes must have the same number of negated components. Nodes in the first piece have two negated components, and nodes in the second have one. The disconnected pieces show that some of the outputs must be produced with separate structures, at least at the last stage.

Each piece of the graph consists of four hexagons that share common edges and lie on the faces of a tetrahedron. As in Fig. 5A, the edge labels show that each hexagon produces six distinct conjunctions. Each hexagon in one piece has a complementary hexagon in the same position in the other piece so that the two hexagons produce 12 distinct conjunctions. For example, the two hexagons drawn in thick lines and their edge labels in boldface show how the 12 outputs represented by the labels are produced in Fig. 1D.

The edge labels show that the outputs that are powers of two (1, 2, 4, 8) can be produced only by the first piece of the graph. Their complements (14, 13, 11, 7) can be produced only by the second piece. The edge labels on the first piece's hexagon in thick lines show that three of the outputs that are powers of two (1, 2, 4) can be produced in two different ways using three alternate cells as excitatory input and the other three as inhibitory input. A check of all the links in the first piece shows this is the fewest cells that can produce the three outputs (except, of course, for the possibility of one less cell as shown in Fig. 5B). The hexagon's remaining outputs (3, 5, 6) can be produced with just three more cells. One of the two possible ways of forming this network is illustrated by the first column in Fig. 1D. All three occurrences of the edge label 8 are circled in Fig. 5C. They are next to the only three nodes that are not connected by one link to the hexagon in thick lines. This means none of the nodes in the hexagon can be used to produce output 8. This output must be produced by a separate structure, at least in the final stage.

The same argument holds for every hexagon in Fig. 5C. Of the four outputs that can be produced only by the piece in which the hexagon lies, each hexagon can produce three with the fewest possible cells, and three more outputs with just three more cells. The fourth output must be produced by a separate structure. This argument shows that two complementary hexagons produce 12 distinct 4-RANCs with the fewest cells. The two remaining 4-RANCs that can be produced according to equations 1 and 2 of Table 2 must be produced with separate structures. Any complementary pair would work as well as another and their n-RANCs have identical architectures, but the hexagons in thick lines were chosen for Fig. 1D because the node names match the simplest possible recursive definition of BC n-RANCs.

It is stated here without proof that the single n-RANC architecture with the minimum number of cells for each output that must be produced by a separate structure is shown in the non-hexagonal columns in Fig. 3. Also stated without proof is that the minimum number of cells that provide input to both the hexagonal and non-hexagonal columns is given by the recursive definition of BC n-RANCs and is illustrated in Figs. 1D, 2.

### Total Connection Length and Cellular Packing Density

**Figure 6:**
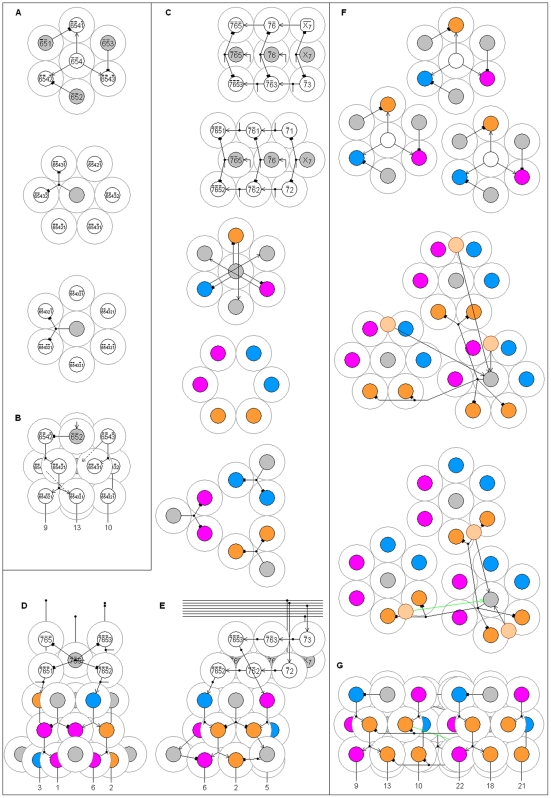
Connection length and cell packing. The connectivity of RANCs suggests an arrangement of cells that nearly optimizes total connection length and cellular packing density. Packing efficiency is illustrated with cell bodies contained in virtual spheres whose diameters are determined by the physiological constraints of necessary separations between cell bodies. Connection length is minimized when two connected cells are in adjacent spheres. The three layers of a typical hexagonal column are shown in A, with the front view of the column in B. Five layers of cells that produce outputs 1–6 in a Complete 7-RANC are shown in C, with the side and front views in D and E. The lower three layers of three columns with shared cells are shown in F and G.

Connection length and cellular packing density will be considered together because both are related to the geometric arrangement of cells. Stains of the neocortex show the bodies of most neighboring neurons are separated by several times the diameter of a cell body. The efficiency of a network's connections and packing can be demonstrated, at least approximately, by placing cell bodies in non-overlapping spheres whose diameters are determined by the constraints of the necessary separations. Although the spheres containing the cells are imaginary, the back hemispheres are made to appear opaque in Fig. 6 so that the three-dimensional aspects of the diagrams are apparent. The basic assumptions in considering optimal cellular arrangements are that 1) the length of a connection between two cells is determined by the distance between the spheres containing them (so that the length is minimized when the spheres are adjacent), and 2) cellular packing density is determined by the packing density of the spheres containing them (so that cellular packing density is maximized when the packing density of their spheres is maximized).

Connection lengths can be minimized by collapsing the exploded views in Figs. 1 and 2 along the lines of connection. This determines the minimum-connection-length spatial arrangement of cells. (Complex connections and the order in which connections are contracted can affect the resulting configuration, but these are not problems for the rather simple connections in BC n-RANCs.) Brain development is apparently consistent with this intuitive approach: Axon elasticity and active force-generating capability are believed to be major factors in the morphogenesis of the brain [3].

All but two hexagonal columns in a BC n-RANC share cells. These networks will be considered first because they determine much of the structure of BC n-RANCs. The various hexagonal columns have some differences in their cells and connections, but the basic argument for the optimal arrangement can be illustrated by a typical column as represented by the second column in Fig. 2. Contracting the connections within the lowest three layers arranges the cells as illustrated in Fig. 6A, B. Top views of the layers are shown in A, and B is the front view.

Minimizing connections between layers requires that the layers be partially nested into adjacent layers. That means the three center spheres must protrude slightly above or below the layers. In Fig. 6B the center spheres protrude equally above and below. This makes a separation of about 0.01 of a sphere's diameter between spheres for the three excitatory connections in the top layer and the two inhibitory connections in the bottom layer. The inputs to the two shared inhibitory cells are partially hidden and are represented by dashed lines in Fig. 6B. The distance between the spheres containing the upper pair of connected cells is about 0.23 of a sphere's diameter. Because the lower center sphere protrudes below its layer, the distance between spheres for the lower connection is about 0.30. The remaining 25 connections are between cells in adjacent spheres. The total distance between spheres containing connected cell pairs is 0.58, an average of less than 0.02 per connected pair.

This configuration does not absolutely minimize the total connection length of the connections within the column, but it clearly comes close. For example, total connection length can be reduced marginally by raising the three center cells slightly. There are only two connections with significant lengths, and a reconfiguration that produces a substantial reduction in either connection will lengthen others even more and reduce packing density. Regardless of slight perturbations that make minor improvements in connection length, the shape will remain essentially a hexagonal column. Although the absolute optimal configuration may not have been found, the only conclusion needed here is that the optimal configuration for lower portions of BC n-RANCs that share inhibitory cells consists of three-layered hexagonal columns similar to the one shown in Fig. 6A, B.

The two hexagonal columns that cannot share inhibitory cells are somewhat different because they must each contain six inhibitory cells. Fig. 6C–E shows part of a BC 7-RANC that produces outputs 1–6 (cf. Fig. 2). Top views of the layers are shown in C, and the side and front views are in D and E, respectively. Since input to three-layered hexagonal columns goes to the uppermost of the three layers, connection length is minimized if the cells that produce the input are placed above this layer. Several of these upper-layer cells, including cells in the single 7-RANC for output 0, are shown as the top two cellular layers in Fig. 6C–E. The reason the cells are placed in two layers will be discussed later.

To improve packing density in the lower layers without affecting connection length, the three inhibitory cells in the second lowest layer of Fig. 2 have been moved to the third lowest layer in Fig. 6C–E. Packing density could be improved somewhat more by moving two of the three inhibitory cells in the lowest layer to the centers of the lower two layers. This would increase connection length slightly. None of these choices affects the conclusions in this article. As in Fig. 6A, B, the absolute optimal configuration may not have been found in Fig. 6C–E, but the main conclusion is that the optimal configuration for the lower cells is a hexagonal column consisting of three layers. Of the 63 connections in this structure, all are between cells in adjacent spheres except for three connections in the third lowest layer (Fig. 6C) and one connection from the top layer to the third lowest layer (Fig. 6E).

Fig. 6F, G shows three hexagonal columns with shared cells (cf. Figs. 2 and 4G). The cost tradeoff in sharing inhibitory cells is fewer cells for longer connections. The total number of connections is the same whether cells are shared or not. Recall that for BC n-RANCs with larger numbers of inputs, many more than the three columns shown in Fig. 6F, G can share just six inhibitory cells. Since the trade is quite asymmetrical, it seems clear that six cells with connection lengths on the order of a millimeter or less should be more cost effective than many cells with shorter connections. Long connections are evidently not prohibitively expensive, as axons of several centimeters and even meter length are common in the nervous system.

To minimize total connection length between columns, columns are clustered in a regular grid of equilateral triangles (Fig. 6F, G). The columns are approximately right circular cylinders, and a regular grid of equilateral triangles is the densest packing configuration for right circular cylinders on a plane. The lower two layers in Fig. 6F show the connections for the two inhibitory cells of the orange module introduced in Fig. 4F. Also shown for each layer are the three cells in the layer above that provide excitatory input to these inhibitory cells. To facilitate clarification of the diagrams, one of these excitatory connections is drawn in green in Figs. 4F, 4G, 6F, and 6G. By the radial symmetry of the layers in Fig. 6F, the other two modules' connections are identical to the orange's.

Finding the densest configuration for packing congruent spheres in three-dimensional Euclidean space was until recently the oldest and most famous unsolved problem in discrete geometry. Attempting to discover the properties of matter in terms of atoms, Johannes Kepler [4] speculated that the densest configuration is the common method of stacking cannonballs. Technically called a face-centered cubic lattice, this is the ordinary pyramidal stack seen today in the fruit sections of grocery stores. The density of this arrangement is 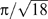
≈0.74. The spheres in the upper two layers of Fig. 6C–E illustrate the optimal pattern, although some of the spheres needed to achieve the maximum density are missing. In a computer-assisted inspection of some 5,000 equivalence classes of possibilities, Kepler's Conjecture was finally found to be correct, along with other equally dense arrangements [5].

The minimum-connection-length columns determined by RANC connectivity do not achieve this optimum packing density, but they are still fairly dense. Packing density is normally computed under the assumption that a packing pattern is repeated without any boundary. For an arrangement of a small number of spheres, the space containing them is not clear. This makes comparing the density of the typical column in Fig. 6B with the optimum density problematical. A lower bound on the density can be computed using the smallest circular cylinder that contains the column. This gives a density of 14/27≈.52, a conservative lower bound since the cylinder contains a considerable amount of empty space around and above and below the column. Rearranging all the spheres of a BC n-RANC to attain the optimal “cannonball” packing would greatly increase the total connection length.

### Number of Layers

Many of the non-hexagonal columns have no connections to other columns in the lower layers. All of the cells in these non-hexagonal columns could therefore be placed in the upper layers without affecting connection lengths within the columns, but the outputs' projections to subcortical regions would be lengthened. In the remaining discussions it will be assumed that the cells shown in the lowest three layers of Fig. 3 are placed in the lowest three layers of BC n-RANCs, and the rest are placed in the upper layers. This assumption will not appreciably affect the conclusions concerning BC n-RANCs.

Useful information about BC n-RANCs can be obtained from a few ratios, one of which is discussed immediately below. The ratios converge asymptotically as n increases. From the recursive definition of BC n-RANCs, close approximations of the limits can be found by numerical methods. The exact values of the limits are not obtained here because some of the limits are convergent series that can only be approximated and because the few inhibitory cells that can be shared in non-hexagonal columns and upper layers were not considered. The given approximations are accurate at least to the number of digits they contain.

It was pointed out earlier that minimizing the total connection length places the cells that produce the input to three-layered hexagonal columns above the columns, but the resulting spatial arrangement of these upper-layer cells may or may not have dense packing. It will now be shown that two layers above the three lower layers are sufficient to contain the upper-layer cells for all n, and two layers are necessary if n≥7. Achieving this packing could mean a few connection lengths are not minimized, such as the connection from the top layer to the third layer in Fig. 6E. This asymmetric trade achieves dense packing of the upper-layer cells at the possible cost of a few slightly longer connections. Few, if any, connections are lengthened because only a few of the cells in the upper layers are connected to the lower three layers. The trade affects the BC n-RANCs' prediction of the number of cellular layers in the neocortex.

No additional cells can be packed into the lowest three layers of hexagonal columns when they are arranged as shown in Fig. 6. The number of additional layers required to contain the upper-layer cells is the ratio of the number of upper-layer cells to the number of cells that can be contained in a layer. The number of upper-layer cells is easily computed from the recursive definition of BC n-RANCs. Seven cells can be placed in each layer above each hexagonal column. For non-hexagonal columns the optimal arrangement of cells has not been considered because for a fairly wide range of possibilities the precise arrangement will not affect the conclusions found here. The diagrams in Fig. 3 suggest two cells can be placed in each layer above each column in the pair of columns shown in Fig. 3A, and three above the lower three layers of each column in Fig. 3B. For all higher values of n, the lower three layers are identical to the lower three layers of Fig. 3C, and four cells can apparently be placed in each layer above these columns.

The ratio of the number of upper-layer cells to the number of cells that can be contained in a layer increases with n. The ratio reaches 1.15 for n = 7 and converges rapidly to about 1.60. That is, two layers in addition to the lower three are sufficient for all values of n, and two are necessary if n≥7. The assumptions of 2, 3, and 4 cells in each layer above each non-hexagonal column can be relaxed considerably. If the average number of cells per layer above each non-hexagonal column is any number between 2 and 6, the ratio determining the number of additional layers converges rapidly to a number between 1 and 2. If all non-hexagonal columns are eliminated, the result is unchanged. In this case, the ratio increases asymptotically to 1.25 and reaches 1.01 at n = 7.

Although the ratio 1.60 (as well as 1.25) means two layers are considerably more than adequate to contain the upper-layer cells in a BC n-RANC, most or all of two layers may be filled for such reasons as redundant cells for error correction and reduced available space in the upper layers if some of a BC n-RANC's possible outputs are not needed. For example, if all of the outputs from one of the hexagonal columns are not needed, eliminating the entire three-layered column reduces the space available in the two layers above that column. Almost all of the upper-layer cells are still needed to produce input to the remaining columns (cf. Fig. 2).

### Modified RANCs

The BC n-RANCs can be modified, simplified, or made more efficient as incomplete n-RANCs in several ways. For example, there may not have been selective pressure to obtain the information in all of the 2^n^ possible outputs. Eliminating some of the single n-RANCs would reduce the lower layers by a number of cells, the number depending on how many of the unneeded outputs share the same columns. The Relative Absorption Model of color vision (RAM) is a modified BC 3-RANC that receives input from three classes of retinal cones and generates neural correlates of the perceptions of red, green, blue, yellow, black, and white [1]. It has six outputs instead of the eight outputs of a BC 3-RANC. The two missing outputs would convey purple and violet information, which is transmitted through the red and blue channels in the RAM. Psychophysical evidence shows that this information is transmitted through the red and blue channels rather than through two separate channels, possibly because there was never selective pressure for the ability to obtain the complete violet and purple information.

Non-hexagonal columns require more cells per output than hexagonal columns. Many non-hexagonal columns can be eliminated under fairly broad conditions. For example, of the 20 outputs that have three negated components in a BC 6-RANC, only 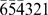 (labeled 7 in Fig. 2) and its complement 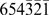 (56) are not produced by a hexagonal column. The input number labels are arbitrary, so if any one of the 20 outputs is not needed, it could be labeled 7 and could be eliminated. If more than one are not needed and any two of the unneeded outputs are complementary, those two could be labeled 7 and 56 and their columns could be eliminated. This would leave all of the remaining single 6-RANCs with three negated components in hexagonal columns.

The single n-RANC that produces the output labeled 0 is a non-hexagonal column that requires n+1 cells added to an otherwise Complete n-RANC. It will be shown that BC n-RANCs average less than 6.1 cells per output. So for n>5, the output 0 requires more additional cells than this average. The response 0 is positive when there is no input. Vision is apparently the only sensory system that produces a perception (black) with no stimulus. For other senses and other uses of RANCs, a response to no input may not be needed. That would eliminate the need for a spontaneously active neuron as well as the other n extra cells in this single n-RANC.

## Results and Discussion

### Combinational Information Processing

The BC n-RANCs go most of the way toward accounting for all of the brain's combinational processing of information. (The outputs of combinational logic functions depend only on the current inputs, not on past values of the inputs.) The rest can be accomplished with as little as a single additional neuron for any given logic function.

The most general function in classical logic is a switching function. For variables that can have the value of 0 or 1, a *switching function* of n variables is an assignment of a 0 or 1 to each of the 2^n^ possible combinations of values of the n variables. For example, the conjunction 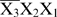 is a switching function of three variables that assigns 1 to the combination of variable values X_3_ = 0, X_2_ = 1, X_1_ = 1 and assigns 0 to the other seven possible combinations. Disjunctions and conjunctions are special cases of switching functions.

A disjunction of conjunctions is also a switching function (e.g., 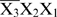 OR 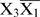). If each of the conjunctions in such a function contains all of the function's variables, with or without negated variables and with no variable appearing more than once, the function is called a *standard* disjunction of conjunctions. (e.g., 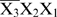 OR 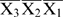 OR 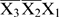 OR 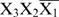). Note that the outputs of a BC n-RANC consist of all possible conjunctions that contain all n of the input variables, with or without negated variables and with no variable appearing more than once. A fundamental theorem in logic design states that every switching function can be expressed as a standard disjunction of conjunctions. This means that any switching function of n variables can be realized with a disjunction of some combination of the outputs of a BC n-RANC.

A disjunction can be constructed with AND NOT gates, but in the brain it might be accomplished more simply by a single neuron with excitatory inputs from all of the components of the disjunction. The natural generalization of property 1 in Table 1 says that a maximum excitation from any one of the neuron's several inputs elicits a maximum response. This minimal assumption implies that for binary inputs of 0 or 1, the neuron's output is the classical logic disjunction of the inputs. For intermediate values of the inputs, this neuron produces a fuzzy logic disjunction if property 3 of Table 1 is also generalized to say the neuron's output is an increasing function of each of the input variables.

### BC n-RANC Efficiency

The BC n-RANCs were defined to be the best of all Complete n-RANCs as measured by the sum of three key cost functions. This definition does not imply the three BC n-RANC costs are economical in comparison to anything besides other Complete n-RANCs. It turns out that BC n-RANCs are quite efficient, not only in the three cost functions that define them but in several other aspects as well.

#### Component requirements

The BC n-RANC component requirements per output are bounded regardless of the number of inputs. As before, the asymptotic limits of the ratio can be approximated by the recursive definition of BC n-RANCs. As n increases, the number of AND NOT gates per output in a BC n-RANC increases asymptotically to about 4.9. Since a neuron functioning as an AND NOT gate requires input from an inhibitory cell, the number of cells per output is somewhat more. As n increases, the number of cells per output (including the output cell itself) increases asymptotically to 6.1. Without sharing of inhibitory cells, the number of cells per output increases asymptotically to 6.6. If all of the less efficient non-hexagonal columns are eliminated, three fourths of the outputs still remain and the number of AND NOT gates per output increases asymptotically to 2.5. In this case, because of the increasing number of columns using each shared cell as n increases, the number of cells per output peaks at 3.625 for n = 4 and 5 and then decreases asymptotically to about 3.3.

The BC n-RANCs can be constructed with electronic components to perform Boolean logic, and the component requirements can be compared with standard electronic Boolean logic arrays. For more than a few inputs, BC n-RANCs require far fewer components. A Boolean AND NOT gate can be constructed with four transistors. Since the number of AND NOT gates per output in a BC n-RANC is bounded at 4.9, a Boolean logic BC n-RANC can be constructed with fewer than 20 transistors per output regardless of the number of inputs. Boolean logic arrays in semiconductor electronics are normally constructed with NAND or NOR gates. A two-input NAND or NOR gate can be constructed with four transistors (the same as an AND NOT gate). In electronic logic arrays, however, conjunctions that have several inputs are normally constructed separately, unlike the n-RANCs that share many AND NOT gates in BC n-RANCs. This means that for standard electronic logic arrays, the component requirement per conjunction increases without bound as the number of inputs increases.

#### Connection length

It was shown that the average connection length in BC n-RANC hexagonal columns is less than two hundredths of the diameter of the spherical space required to contain cell bodies, where connection length is measured by the distance between spheres. This is a fortuitous feature of BC n-RANCs because minimum-connection-length configurations do not in general imply short connections. This can be demonstrated with a simple counterexample. Consider a set of congruent spheres in three-dimensional space with each sphere connected to every other sphere, and with the spheres arranged in a minimum-connection-length configuration. Both the average connection length and the total connection length are unbounded, increasing functions of the number of spheres.

#### Packing density

It was shown with a conservative estimate that the packing density in hexagonal columns is more than two thirds of the maximum possible density. The fairly dense packing is largely a result of the hexagonal shape and nested layers of the columns as determined by the minimum-connection-length configuration. Like the short connections, dense packing is a fortuitous feature of BC n-RANCs because minimum-connection-length configurations do not in general imply high density. This can be demonstrated with a minor modification of the hexagonal column of Fig. 6A, B. Consider an equilateral triangular cylinder. The cylinder walls are composed of congruent spheres arranged as a few layers of Kepler's optimal “cannonball” stack. Each sphere is connected to its adjacent neighbors, so the total connection length is zero. The triangular cylinder is the only minimum-connection-length configuration because any rearrangement of spheres, such as folding the walls, would change the optimal “cannonball” packing and therefore lengthen some of the connections. Keeping the size of the triangular cylinder fixed, walls composed of sufficiently small spheres can make the packing density of the cylinder arbitrarily close to zero.

#### Modular architecture

The BC n-RANCs' modular architecture is largely a result of the recursive definitions for RANCs and BC n-RANCs. Modular architecture greatly simplifies the assembly process.

#### Number of component types

Only two types of components are required for a BC n-RANC. The first is a component that provides a spontaneous and continuous high output signal. Only one component of this type is needed. In the brain, a neuron that fires spontaneously and continuously can serve as this component. In electronic logic circuitry, this function can be provided by the power supply. The second component is an AND NOT gate that allows continuous as well as discrete input values and provides a response that is measure of the difference between the two input values.

#### Fuzzy logic

It was shown above that for more than a few inputs, BC n-RANCs composed of transistors perform Boolean logic with far fewer components than standard electronic logic arrays. For fuzzy logic, BC n-RANCs composed of fuzzy AND NOT gates are also much more efficient than standard electronic systems. By the properties of Table 1, a single neuron together with an inhibitory neuron provides a measure of the difference between the inputs in a single operation. Conventional computational systems that encode information in discrete zeros and ones would require many components and many operations to carry out this fuzzy AND NOT computation. In addition, by the RANC interval measure property, RANCs composed of fuzzy AND NOT gates perform fuzzy logic [2]. Even if a fuzzy AND NOT gate were to be constructed with electronic components, connecting them to produce fuzzy logic has apparently not been demonstrated with any architecture other than RANCs.

#### Nonlinearities

Nonlinearities in the AND NOT function do not have serious adverse effects on the system. An accurate measure of the input difference is not necessary to generate useful fuzzy logic since such logic deals with largely subjective assignments of truth values. Useful fuzzy logic follows from the RANC interval measure property because each RANC output increases as the length of the interval it measures increases. That is, a higher degree of truth in the conjunction is reflected in a higher degree of truth in the RANC's measure of the truth value.

#### Information

In spite of possible nonlinearities in the AND NOT function, BC n-RANC outputs retain all of the information in the inputs [2].

#### Layers

The BC n-RANCs have a limited number of layers. It was shown that five layers are sufficient for any n.

#### Connection directions

Since nearly all connections are either within layers or between adjacent layers and perpendicular to the layers, the architecture is conducive to a three-dimensional, layered construction. The simple connections greatly simplify the assembly process.

#### Computation time

Although the number of BC n-RANC outputs grows exponentially with the number of inputs (2^n^ conjunctions of n propositions), computation time grows linearly. The total time required to compute all 2^n^ outputs is the time it takes for a signal to pass through n−1 AND NOT gates. This property follows by induction from the recursive definition of BC n-RANCs.

Whether or not conjunctions can be formed with fewer AND NOT gates using logic circuit architectures other than RANCs, i.e., not according to the logic identities in Table 2, has not been considered here. It appears to be unlikely that any architecture could significantly improve on either ratio of 4.9 AND NOT gates per conjunction or, in the case of logic circuits composed of neurons, 6.1 cells per conjunction. Although BC n-RANCs appear to minimize, or nearly minimize, the number of AND NOT gates required for logical conjunctions constructed under any architecture, the accuracy of this conjecture is not essential to the utility of the model. In any case BC n-RANCs are quite efficient and accurately account for several significant properties of neural processing and cortical structure.

The effect of multiple excitatory and inhibitory inputs on a single neuron's function as a logic gate has also not been considered, not only because of the complexity of such logic systems but because no definitive properties comparable to those in Table 1 are known about a neuron's output as a function of several inputs. As was pointed out previously [2], multiple connections typical of neurons do not necessarily mean all networks in the brain operate fundamentally differently from RANCs. For a variety of reasons, such as the cells that perform disjunctions and the shared cells that were discussed earlier, or redundancies and other error-correcting mechanisms, neurons in the brain may have several connections while performing the same functions as RANCs in essentially the same ways. The purpose of RANCs is to show that logic circuits composed of neurons can perform known brain functions. Actual networks in the brain could be organized like these minimal networks in principle while being more elaborate in the details.

### RANC Predictions

The BC n-RANCs predict several important characteristics of the anatomical structure and physiological organization of the neocortex. These BC n-RANC properties are genuine predictions because BC n-RANCs were defined to be specific logic circuits – conjunctions – with architectures that exploit basic neural capabilities and optimize certain cost functions; they were not designed to fit available data about the brain.

To avoid confusion it should be noted that these predictions do not depend on the BC n-RANC efficiencies discussed earlier. The BC n-RANCs make accurate predictions regardless of how cost effective they are in comparison to anything besides other Complete n-RANCs. The efficient properties of the logic circuits are interesting and may say something important about the brain and applications in electronic circuits, but they are irrelevant to the predictions given here.

#### Layers

The neocortex is a thin lamina, and it is organized in distinct layers of cells. The most typical form consists of six layers. The outer layer is acellular, consisting mainly of dendrites from lower layer cells and axons that lie within the layer [6]. The section on the number of layers showed that BC n-RANCs are layered, five cellular layers are sufficient for all n, and five cellular layers are necessary if n≥7.

#### Corticocortical projections

Projections from one part of the neocortex to another come mainly from neurons in layers II and III (the two outermost cellular layers) [7]. It was shown that nearly every cell in the upper two cellular layers of a BC n-RANC provides input to more than one column. Except for small values of n, at least some of these columns would be widely separated. In contrast, nearly all connections in the lower three BC n-RANC layers are within columns.

#### Subcortical projections

Projections from the neocortex to subcortical regions come mainly from layers V and VI (the two innermost layers) [7]. Responses in the lower RANC layers contain the most information because they are conjunctions of the most inputs. Signals conveying the most information would be the most useful and therefore would be the most likely to be projected to other parts of the brain.

#### Connection directions

Weigert stains for myelinated fibers show nearly all axons run parallel or perpendicular to the layers [8]. Only a few axons run diagonally for short distances, no more than the thickness of one layer. The restrictions on connection directions are not an insignificant design feature of the brain, considering the possible connections among nodes in three-dimensional space. Fig. 6 shows that nearly all of the connections in the lower three layers of a BC n-RANC are horizontal or vertical. The only diagonal connections are the excitatory inputs from one column to shared inhibitory cells in another column. These connections extend only from one layer to the layer below (Fig. 6F, G).

#### Connection directions – outer layers

Weigert stains also show nearly all axons in the outer two layers (including the acellular layer) and much of the third layer are parallel to the layers. Although the minimum-connection-length arrangement in a BC n-RANC's upper two cellular layers varies with the number of inputs, an exact optimal arrangement is not necessary to see that these layers are consistent with mostly horizontal connections. The basic argument is simply that nearly all of the connections in the upper two layers are between cells within those layers. This property follows from the recursive definition of BC n-RANCs and is illustrated in Fig. 2. The configuration in Fig. 6C–E minimizes the total connection length for several of the cells in the upper two layers of a BC 7-RANC. All of the excitatory connections are horizontal except for the few inputs to, and outputs from, the two layers.

#### Placement of cell types

Inhibitory cells are located in all cellular layers [6]. Fig. 6 shows that BC n-RANCs have inhibitory cells in all layers.

#### Proportion of cell types

**Figure 7:**
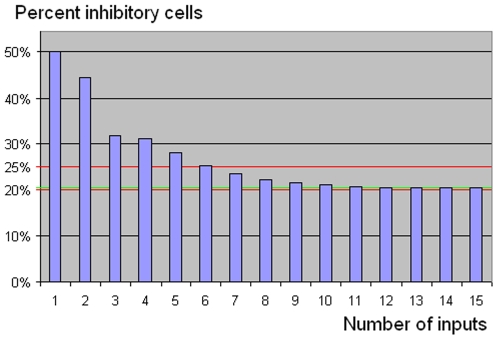
Inhibitory cells in BC n-RANCs. The graph shows the BC n-RANC's percentage of inhibitory cells as a function of the number of inputs n. Input cells are not counted as part of the network. In the cortex, 20 to 25% of the neurons are inhibitory. This agrees well with BC n-RANCs, which have between 20 and 25% inhibitory neurons for all n≥7. The proportion is about 23.6% for n = 7. As n increases, the proportion decreases asymptotically to about 20.4%, shown in green in the graph.

Inhibitory cells constitute 20 to 25% of the neurons in the neocortex [6]. This closely matches BC n-RANCs. The graph in Fig. 7 shows the percentage of inhibitory cells in BC n-RANCs as a function of n. For n≥7, all BC n-RANCs have between 20 and 25% inhibitory cells. The percentage is 23.6% for n = 7, and as n increases the percentage decreases asymptotically to 20.4%. Without sharing of inhibitory cells, the percentage of inhibitory cells is significantly greater, decreasing asymptotically to 26.4%. So RANCs that minimize resource requirements by sharing inhibitory cells agree with empirical evidence in the brain.

If all of the less efficient non-hexagonal columns are eliminated, such modified BC n-RANCs still predict the percentage of inhibitory cells to be between 20 and 25% although the match is not quite as good. The percentage decreases asymptotically to 24.5%, first dropping below 25% for n = 12 at 24.8%. So BC n-RANCs with at least some non-hexagonal columns are more consistent with the neocortex. Constructing BC n-RANCs with the absolute minimum number of cells also affects the prediction of the percentage of inhibitory cells. The percentage decreases asymptotically to 25.0% and is not less than 25% for any value of n. So retaining more than the absolute minimum number of cells to maintain functionality and modularity is also more consistent with the neocortex.

#### Computational modules

Information is processed in small columns of neurons connected across the cortical layers and spanning all layers [6]. It was shown earlier that BC n-RANCs exhibit all of these properties, which are illustrated in Figs. 1, 2, and 6.

#### Information transformation

Information is transformed in each layer of the neocortex while passing through the columns in series and in parallel [6]. These properties follow from the RANC definition in Table 2 and the columnar structure of BC n-RANCs. The properties are illustrated by the example data in Figs. 1 and 2, especially Fig. 2.

#### Suppressed regions

Active regions in the neocortex are surrounded by neurons that are inhibited below the resting level [9]. In a BC n-RANC, the phenomenon is predicted by the efficient use of inhibitory cells to inhibit more than one cell and by the minimum-connection-length arrangement of such cells. Suppression below the resting level occurs when a cell has inhibitory input with no excitatory input. In BC n-RANCs such suppression is most prevalent in sets of columns that share inhibitory cells. These columns are clustered together to minimize connection length between them, no more than one of the columns is active at any time, and each active shared cell suppresses two cells below the resting level in every inactive column. Thus the clustering of columns with shared cells predicts active regions surrounded by neurons inhibited below the resting level. In Fig. 2, the example inputs produce several such suppressed cells that can be found by their inhibitory input in red with no excitatory input in green. Fig. 2 shows only two columns with shared cells. Larger sets of columns with shared cells would exhibit more neurons inhibited below the resting level.

### Few Inputs

With small values of n, BC n-RANCs can account for the structure of some other portions of the nervous system that have fewer layers than the neocortex. It has been noted previously that a color vision model composed of modified BC 3-RANCs that have inputs from three classes of photoreceptors predicts that the retina consists of three main layers of cells, where the photoreceptors make up one of the layers [1]. Small n n-RANCs also account for fewer layers in portions of the brain that are phylogenetically older than the neocortex, such as the hippocampus and cerebellar cortex that both have three cellular layers.

It was shown earlier that if n≥7, BC n-RANCs predict both the five cellular layers and the 20–25% inhibitory cells of the neocortex. The restriction on n is not necessary for all BC n-RANCs, however, to account for these characteristics of the brain. If some RANCs in the neocortex have few inputs, RANCs with more inputs contribute more heavily to any average by their size and possibly by their number. Since the number of cells in a BC n-RANC approximately doubles with each additional input, the weighted contribution to the percentage of inhibitory cells or average number of layers also doubles. This implies that not even a majority of RANCs with seven or more inputs is necessary to achieve 20–25% inhibitory cells and an average of five cellular layers. Moreover, since the neocortex is apparently responsible for more complex information processing than the rest of the brain, and because RANC outputs with more inputs convey more information than those with few inputs, RANCs with many inputs may be expected to be more abundant than RANCs with few inputs.

### Summary and Conclusion

Two previous articles presented Recursive AND NOT Conjunctions (RANCs), a family of explicit neural logic circuits that generate detailed neural correlates of known psychophysical phenomena. Here it was shown that certain architectures for these networks optimize the total cost of the number of cells, connection length, and cellular packing density. These Best Complete n-RANCs provide the foundation for all of the brain's combinational processing of information, are markedly efficient in the three defining cost functions as well as several others, produce correlates of electrophysiological brain phenomena, and predict major aspects of the anatomical structure and physiological organization of the neocortex. Electronic implementations of BC n-RANCs may be more efficient than current electronic logic arrays in generating both Boolean and fuzzy logic.

The only types of components required are one that provides a spontaneous and continuous output signal and a logical AND NOT gate that allows continuous as well as discrete input values and provides a response that is measure of the difference between the two input values. A neuron performs this fuzzy AND NOT function much more efficiently than conventional computational systems that encode information in discrete zeros and ones. No more than 4.9 AND NOT gates per output are required regardless of the number of inputs, and no more than 6.1 cells per output. The architecture is modular and recursive and is conducive to a three-dimensional, layered configuration, with no more than five cellular layers required regardless of the number of inputs. Connections that are either within layers or perpendicular to the layers simplify the assembly process. Although the number of outputs grows exponentially with the number of inputs, the time required to compute all outputs grows linearly.

The BC n-RANCs account for significant local properties of the neocortex's structural organization, including the five cellular layers, axons extending mainly parallel or perpendicular to the layers, axons in the outer layers mainly parallel to the layers, projections from one part of the neocortex to another coming mainly from neurons in layers II and III, projections to subcortical regions mainly in the lower layers, and inhibitory cells located in all cellular layers and constituting 20 to 25 percent of the neurons. The architecture also predicts local electrophysiological phenomena of the neocortex, including information processed in small columns of neurons connected across the layers, information transformed in each layer while passing through the columns in series and in parallel, and active regions surrounded by neurons that are suppressed below the resting level.
